# G.I.S. Surveillance of Chronic Non-occupational Exposure to Heavy Metals as Oncogenic Risk

**DOI:** 10.3934/publichealth.2016.1.54

**Published:** 2016-02-29

**Authors:** Ioan Stelian Bocşan, Irina Brumboiu, Tudor Călinici, Mariana Vlad, Cecilia Roman, Ioana Brie, Mihaela Lucia Ponta

**Affiliations:** 1 The Iuliu Haţieganu University of Medicine and Pharmacy, Cluj-Napoca; 2 The Professor Dr. Iuliu Moldovan Institute of Public Health Cluj-Napoca; 3 ICIA Cluj-Napoca; 4 The Professor Dr. Ion Chiricuţă Oncologic Institute Cluj-Napoca; 5 The Babeş-Bolyai University, Cluj-Napoca; Romania

**Keywords:** heavy metals, non-occupational exposure, oncogenic effects, GIS surveillance

## Abstract

**Introduction:**

The potential oncogenic effect of some heavy metals in people occupationally and non-occupationally exposed to such heavy metals is already well demonstrated. This study seeks to clarify the potential role of these heavy metals in the living environment, in this case in non-occupational multifactorial aetiology of malignancies in the inhabitants of areas with increased prevalent environmental levels of heavy metals.

**Methods:**

Using a multidisciplinary approach throughout a complex epidemiological study, we investigated the potential oncogenic role of non-occupational environmental exposure to some heavy metals [chrome (Cr), nickel (Ni), copper (Cu), zinc (Zn), cadmium (Cd), lead (Pb) and arsenic (As)—in soil, drinking water, and food, as significant components of the environment] in populations living in areas with different environmental levels (high vs. low) of the above-mentioned heavy metals. The exposures were evaluated by identifying the exposed populations, the critical elements of the ecosystems, and as according to the means of identifying the types of exposure. The results were interpreted both epidemiologically (causal inference, statistical significance, mathematical modelling) and by using a GIS approach, which enabled indirect surveillance of oncogenic risks in each population.

**Results:**

The exposure to the investigated heavy metals provides significant risk factors of cancer in exposed populations, in both urban and rural areas [***χ^2^*** test (*p* < 0.05)]. The GIS approach enables indirect surveillance of oncogenic risk in populations.

**Conclusions:**

The role of non-occupational environmental exposure to some heavy metals in daily life is among the more significant oncogenic risk factors in exposed populations. The statistically significant associations between environmental exposure to such heavy metals and frequency of neoplasia in exposed populations become obvious when demonstrated on maps using the GIS system. Environmental surveillance of heavy metals pollution using GIS should be identified as an important element of surveillance, early detection, and control of neoplastic risks in populations, at the level of a single locality, but even on a wider geographical scale.

## 1. Introduction

It is more than a decade since the search for correlations between chronic, non-occupational exposure of human populations to certain heavy metals, the health status (morbidity) experienced by these populations, and their risk of development of cancer became one of the EÙs priorities in the context of the European *Environment and Health Action Plan 2004–2010 (EHAP)* Programme [1].

Despite the fact that the aetiology of cancer is multifactorial, the known potential oncogenic effects of some heavy metals—among many other harmful effects—in subjects who are occupationally and non-occupationally exposed to such heavy metals, has been well demonstrated [2]–[9].

There are many relevant studies of the potential carcinogenic roles some heavy metals could play, particularly in occupationally exposed people. The role of chronic exposure to heavy metals in non-occupationally exposed populations is less clear. This paper presents some outcomes of a three-year study of this topic, in two counties in North-Western Romania, on randomly selected representative samples of exposed and non-exposed subjects from within the general population. The level of heavy metals in that environment were studied cross-sectionally (in soil, air, drinking water, common foods), and the results were compared with historical results from similar studies performed during the previous three decades by the same laboratories. Biological tests were performed in order to establish the impact on peoplès health of environmental exposure to heavy metals. All tests were finally correlated with the health status of the populations of the two regions. The preliminary results presented in this paper show significant differences to the extent of non-occupational exposure to heavy metals of environmental origin in the two selected areas.

## 2. Materials and methods

In the frame of the multifactorial causality of cancers, using a multidisciplinary approach through a complex epidemiological study, we investigated the potential oncogenic role of non-occupational environmental exposure to some heavy metals, in population living in areas with different prevalent environmental levels (high vs. low) of heavy metals. In other words, this study seeks to clarify the potential role of such heavy metals in the living environment in the context of non-occupational multifactorial aetiology of malignancies in areas with increased environmental levels of heavy metals.

The study was performed during the period 2006–2008 in the North-Western part of Romania, covering a geographical area containing naturally existing high levels of heavy metals (Maramures County) in its environment, to be compared with a control area having much lower naturally existing levels of heavy metals as pollution (Cluj County). The levels of heavy metal ambient pollution and their correlation with the incidence of malignancies were analysed in exposed humans living in the polluted study area (Maramures County), as compared to the results obtained in the control less polluted area (Cluj County). We analysed as the different potential biomarkers the levels of chrome (Cr), nickel (Ni), copper (Cu), zinc (Zn), cadmium (Cd), lead (Pb) and arsenic (As) in soil, drinking water, and food, as environmental elements. The results were correlated and interpreted using the GIS mapping method.

In this paper, we focused on the following objectives:

- Evaluation of exposure to selected heavy metals in urban and rural populations in designated areas;
- Evaluation of the potential use of GIS as a surveillance tool to estimate the impact of environmental factors on the health status of populations.

Levels of exposure could be calculated based on identification of the exposed populations, of the critical elements of ecosystem, and as according to the ways of identifying the means of exposure.

For data collection we used a questionnaire (which included questions on general information, personal data, data on the households of subjects, exposure, health module, biomarkers, food, and chronic conditions having impact on nutritional status). The questionnaires were completed, not by the subjects interviewed, but by specialists in our institution who had been trained to implement these questionnaires. Questionnaires were originally validated on a sample of subjects. The research and questionnaire used were approved by the Ethics Committee of the University of Medicine and Pharmacy. The questionnaires were used only for subjects who had signed an informed consent form.

The evaluation of risks of cancer incidence associated to chronic non-occupational exposure at low levels to heavy metals, individually or in combinations, involved the following objectives:

- Establishing the history of pollution in the areas of interest.
- Determination of the concentration of heavy metals (Pb, Cu, Cd, Zn, As, Cr) in soil, drinking water, food and biological samples obtained from the exposed subjects living in the area studied, and from the control subjects unexposed to heavy metal pollution.
- Correlating the available epidemiological, environmental and geographical data, stored in databases.

The evaluation of the role that chronic exposure to heavy metals in food and drinking water might have had in populations exposed or unexposed to low levels of these metals, and with /without malignancies, was performed by monitoring the intake of heavy metals in food and water, as determined by laboratory analysis of local samples of food and drinking water (data to be published). All cases of malignancies reported to/by public health authorities were included, not making any specific relationship between any given heavy metal and any particular neoplasia.

## 3. Results

The results were interpreted both epidemiologically (causal inference, statistical significance, mathematic modelling) and using the GIS approach. GIS maps were constructed for both investigated areas, combining both medical and environmental investigated parameters, allowing indirectly the surveillance of oncogenic risk in those populations.

**Figure 1.**
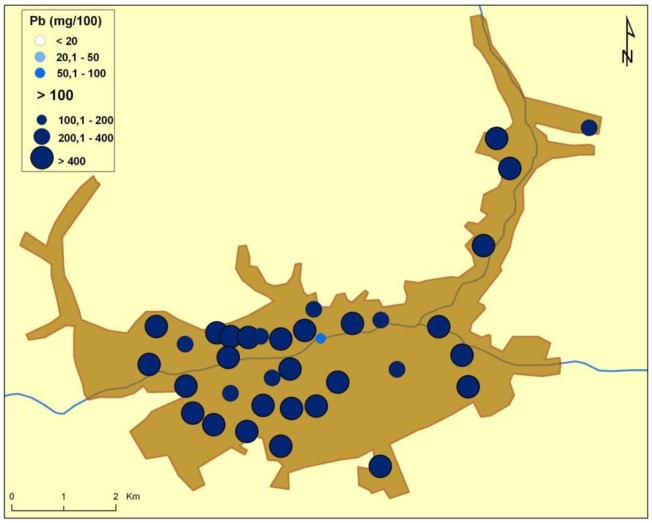
Lead environmental pollution in Baia Mare.

**Figure 2.**
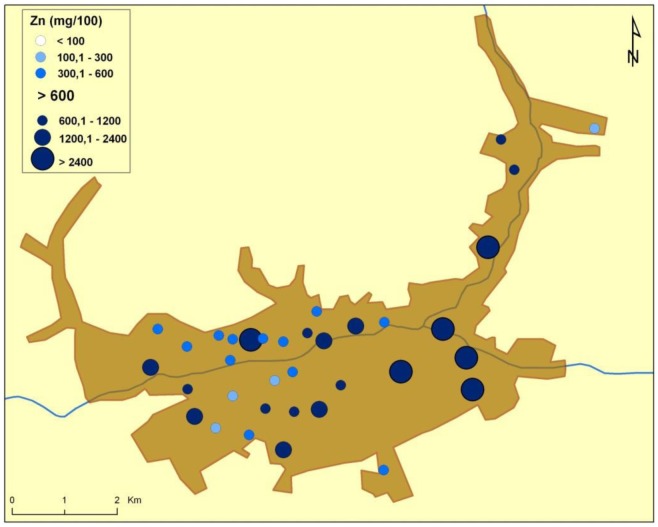
Zinc environmental pollution in Baia Mare.

**Figure 3.**
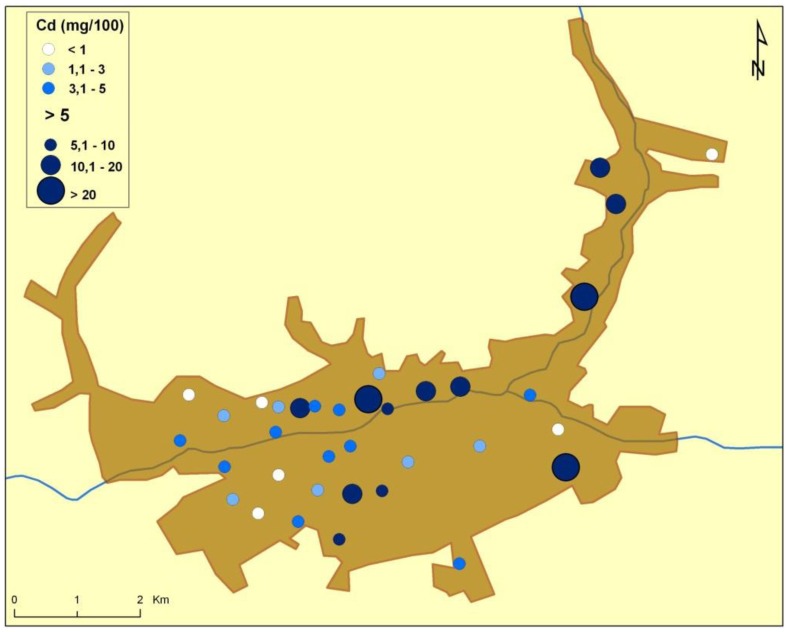
Cadmium environmental pollution in Baia Mare.

**Figure 4.**
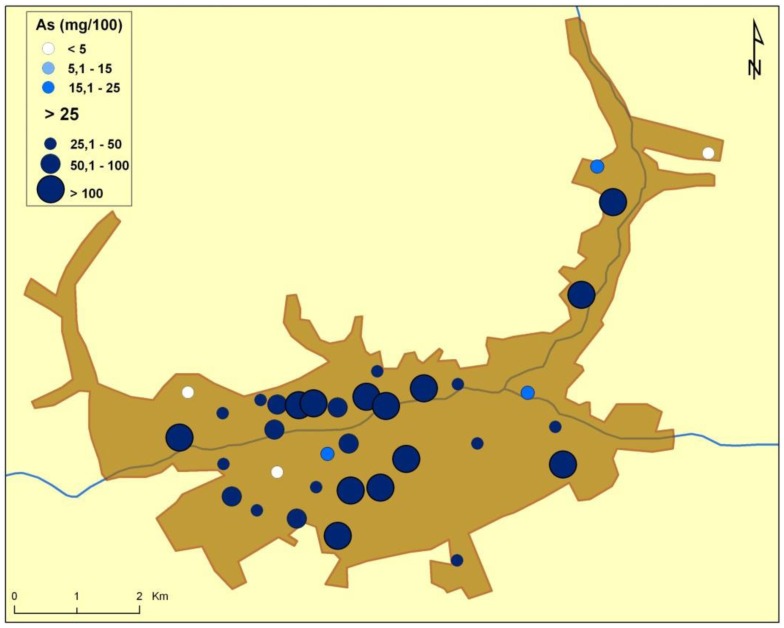
Arsenic environmental pollution in Baia Mare.

**Figure 5.**
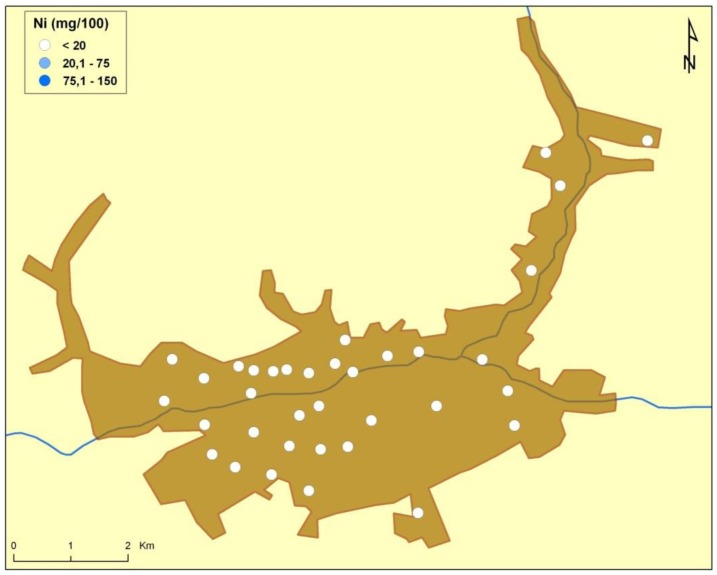
Nickel environmental pollution in Baia Mare.

**Figure 6.**
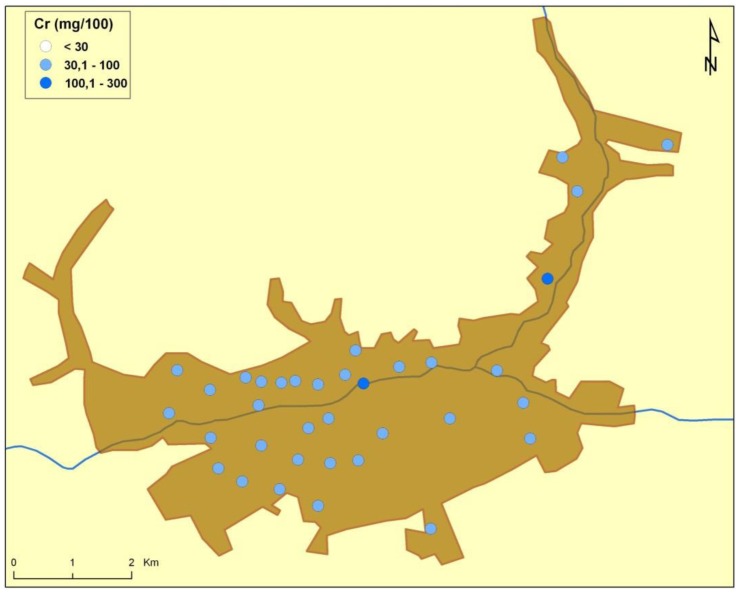
Chrome environmental pollution in Baia Mare.

**Figure 7.**
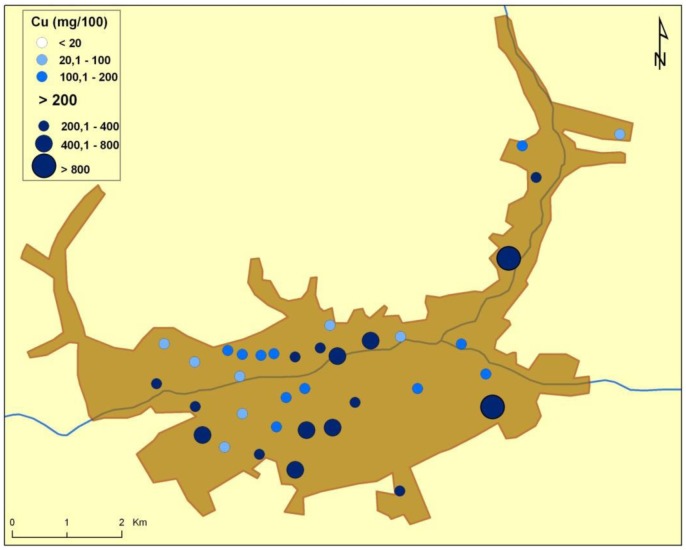
Copper environmental pollution in Baia Mare.

There are naturally-occurring higher levels of the investigated microelements in the environment surrounding Baia Mare (the capital city of Maramureş County) area than can be found in Cluj County, as shown in the following GIS maps (Figure 1–7).

**Figure 8.**
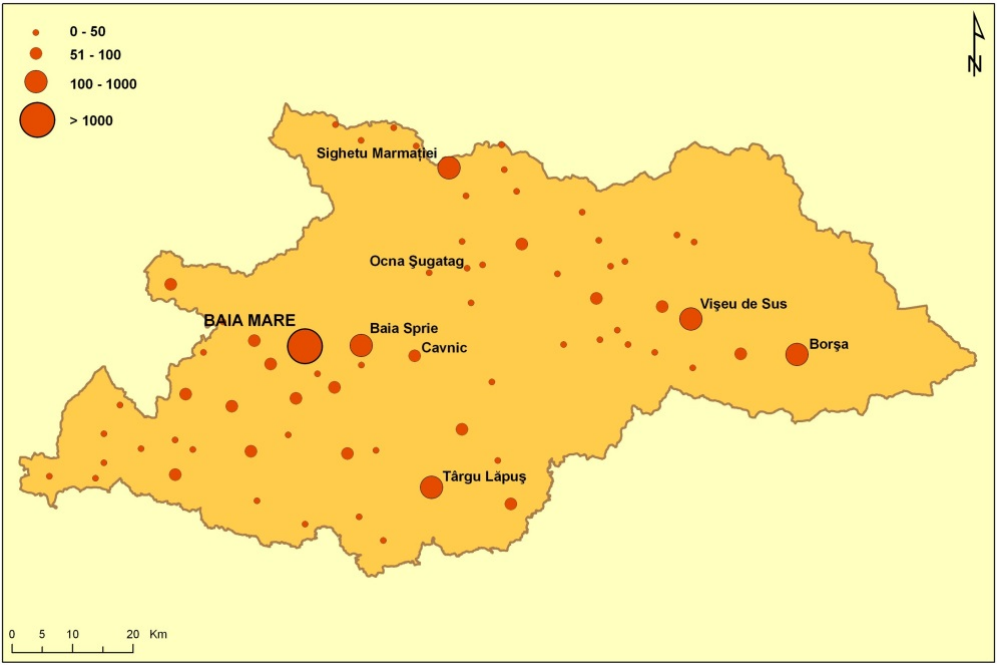
Cancer prevalence in Maramures County.

**Figure 9.**
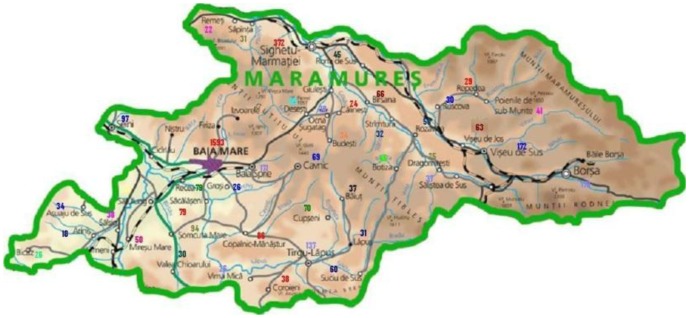
Cancer prevalence in Maramures County.

**Figure 10.**
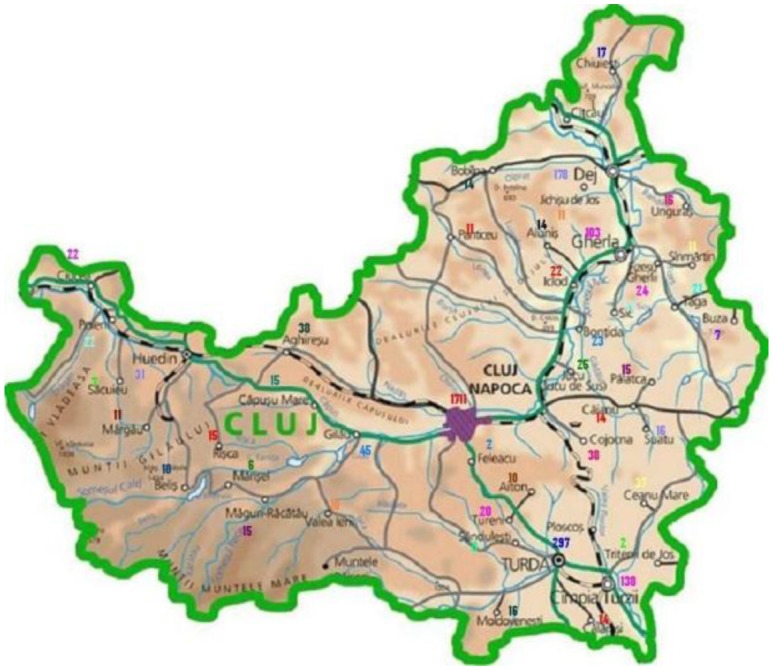
Cancer prevalence in Cluj County.

The prevalence of cancers in the inhabitants of the two areas is higher in the heavier polluted area (Maramureş county) (Figure 8, 9, 10).

The ***χ^2^*** test proved (*p* < 0.05) that exposure to heavy metals is associated with a significantly increased risk of cancer in exposed populations, both in urban and rural areas.

The RISCANSIM software that has been created (by TC) allows the user to create personal scenarios of heavy metals exposure. The user has the possibility to set the concentration of different heavy metals, and the application can estimate the prevalence of cancers and of the genetic disorders for the given scenario.

## 4. Discussion

The oncogenic effects of environmental non-occupational exposure to heavy metals in human populations are well-known and accepted [9],[10]. To our knowledge, this is the first and only study investigating the role of environmental non-occupational exposure to heavy metals in oncogenesis in exposed human populations in North-Western Romania.

The major objectives to be considered when evaluating the exposure to in-taking pollutants were:

- the source of investigated chemical agent;
- means of exposure;
- measuring / estimating concentrations and duration of exposure;
- defining the exposed population;
- the integral analysis of exposure.

The existing industrial sources of pollution (in Maramures County) have a certain influence on the depreciation of the quality of the environment (soil included). The appropriate and accurate evaluation of this phenomenon could be made only if the natural background geochemical composition of the soil in the area is known. The concentrations of available metals in soil constitute mainly the toxic fraction (having influence on plants and underground waters). The maximal values accepted for heavy metals in soil refer exclusively to total concentrations and vary from country to country.

We did not intend to assess individually the relationship of each heavy metal with cancer incidence, on account of the simultaneous presence and effects of other non-occupational exposures which the general population experiences in each area. The relationship between the levels of environmental pollution and oncogenic risks of exposed populations are well known, even if some opinions are divergent [11]–[13]. Such relationships could be monitored and evaluated using modern techniques such as GIS and computer assisted simulation; if the surveillance of both environment and health is of appropriately high quality, GIS mapping is a powerful tool in identifying the possible sources of pollutants [14]–[18].

## 5. Conclusions

Despite the multifactorial etiological factors influencing the incidence of oncological conditions, the role of heavy metals in the non-occupational environment aspects of our daily lives appears to be important but – unfortunately – is insufficiently studied and understood.

Chronic exposure to some heavy metals is one of the relevant oncogenic risk factors in exposed populations.

Statistical significant associations between environmental exposure to selected heavy metals and incidence of neoplasia in exposed populations has been clearly demonstrated, and could be represented on maps using GIS systems.

The environmental surveillance of heavy metals pollution using GIS could be an important element in the surveillance, early detection, and control of neoplastic risks in populations, including when applied more generally than when applied only in one locality.

A high priority must be a determination to enlarge the spectrum of health-related variables to be used as indicators for environmental factors (air, drinking water, soil).

New regulations would have to include maximal admitted values for the concentration of available metals in soil that represent the potential toxic fraction for plants which might enter the food chain.

## References

[1] European Environment and Health Strategies (EHAP) 2004-2010 Programme.

[2] Fasinu P, Orisakwe OE (2013). Heavy metal pollution in sub-Saharan Africa and possible implications in cancer epidemiology. Asian Pac J Cancer Prev.

[3] Tabrez S, Priyadarshini M, Priyamvada S (2014). Gene-environment interactions in heavy metal and pesticide carcinogenesis. Mutat Res Genet Toxicol Environ Mutagen.

[4] Åkesson A, Barregard L, Bergdahl IA (2014). Non-renal effects and the risk assessment of environmental cadmium exposure. Environ Health Perspect.

[5] Adams SV, Quraishi SM, Shafer MM (2014). Newcomb PA: Dietary cadmium exposure and risk of breast, endometrial, and ovarian cancer in the Women's Health Initiative. Environ Health Perspect.

[6] Hartwig A (2013). Cadmium and cancer. Met Ions Life Sci.

[7] Nagata C, Nagao Y, Nakamura K (2013). Cadmium exposure and the risk of breast cancer in Japanese women. Breast Cancer Res Treat.

[8] Kossowska B, Dudka I, Gancarz R (2013). Application of classic epidemiological studies and proteomics in research of occupational and environmental exposure to lead, cadmium and arsenic. Int J Hyg Environ Health.

[9] Bacquart T, Frisbie S, Mitchell E (2015). Multiple inorganic toxic substances contaminating the groundwater of Myingyan Township, Myanmar: arsenic, manganese, fluoride, iron, and uranium. Sci Total Environ.

[10] Liu X, Song Q, Tang Y (2013). Human health risk assessment of heavy metals in soil-vegetable system: a multi-medium analysis. Sci Total Environ.

[11] Verougstraete V, Lison D, Hotz P (2003). Cadmium, lung and prostate cancer: a systematic review of recent epidemiological data. J Toxicol Environ Health B Crit Rev.

[12] Yuan X, Wang J, Shang Y, Sun B (2014). Health risk assessment of cadmium via dietary intake by adults in China. J Sci Food Agric.

[13] Saleem M, Iqbal J, Shah MH (2014). Non-carcinogenic and carcinogenic health risk assessment of selected metals in soil around a natural water reservoir, Pakistan. Ecotoxicol Environ Saf.

[14] Liu X, Wu J, Xu J (2006). Characterizing the risk assessment of heavy metals and sampling uncertainty analysis in paddy field by geostatistics and GIS. Environmental Pollution.

[15] Lee CS, Li X, Shi W (2006). Sharon Ching-nga CheungMetal contamination in urban, suburban, and country park soils of Hong Kong: A study based on GIS and multivariate statistics. Environmental Pollution.

[16] Zhang C (2006). Using multivariate analyses and GIS to identify pollutants and their spatial patterns in urban soils in Galway, Ireland. Environmental Pollution.

[17] Facchinelli A, Sacchi' E, Mallen L (2001). Multivariate statistical and GIS-based approach to identify heavy metal sources in soils. Environmental Pollution.

[18] Colak EH, Yomralioglu T, Nisanci R (2015). Geostatistical analysis of the relationship between heavy metals in drinking water and cancer incidence in residential areas in the Black Sea region of Turkey. J Environ Health.

